# Bioactive Potential of Balkan *Fomes fomentarius* Strains: Novel Insights into Comparative Mycochemical Composition and Antioxidant, Anti-Acetylcholinesterase, and Antiproliferative Activities

**DOI:** 10.3390/microorganisms13061210

**Published:** 2025-05-26

**Authors:** Milena Rašeta, Marko Kebert, Diandra Pintać Šarac, Jovana Mišković, Sanja Berežni, Ágnes Erika Kulmány, István Zupkó, Maja Karaman, Suzana Jovanović-Šanta

**Affiliations:** 1 Department of Chemistry, Biochemistry and Environmental Protection, Faculty of Sciences, University of Novi Sad, 21000 Novi Sad, Serbiasuzana.jovanovic-santa@dh.uns.ac.rs (S.J.-Š.); 2 Institute of Lowland Forestry and Environmental Protection, 21000 Novi Sad, Serbia; kebertmarko@gmail.com; 3 Department of Biochemistry, Faculty of Medicine, University of Novi Sad, 21000 Novi Sad, Serbia; diandra.pintac@mf.uns.ac.rs; 4 ProFungi Laboratory, Department of Biology and Ecology, Faculty of Sciences, University of Novi Sad, 21000 Novi Sad, Serbia; jovana.maric@dbe.uns.ac.rs (J.M.); maja.karaman@dbe.uns.ac.rs (M.K.); 5 Institute of Pharmacodynamics and Biopharmacy, University of Szeged, 6720 Szeged, Hungary; kulmany.agnes@gmail.com (Á.E.K.); zupko.istvan@szte.hu (I.Z.)

**Keywords:** Balkan region, bioactive compounds, *Fomes fomentarius*, neuroprotective agents, oxidative stress, phenolics, polyamines

## Abstract

*Fomes fomentarius* (L.) Fr. has been valued since the 15th century across Europe, including the Balkan region, for its medicinal and traditional uses such as tinder production, spiritual practices, wound healing, and hemostasis. This study analyzes three Balkan strains, focusing on micro- and macroelements, polyamines (PAs), and phenolic compounds in hot water (H_2_O), chloroform (CHCl_3_), hydroethanolic (EtOH), and hydromethanolic (MeOH) extracts. Micro- and macroelements were determined using atomic absorption spectrophotometry (AAS), while PAs were quantified using high-performance liquid chromatography with fluorescence detection (HPLC-FD). Phenolic profiles were determined using liquid chromatography–mass spectrometry (LC-MS/MS), with total phenolic content (TPC) assessed using the Folin–Ciocalteu method. Antioxidant activity was evaluated via DPPH, ABTS, NO scavenging, lipid peroxidation inhibition, and FRAP assays, alongside anti-acetylcholinesterase and antiproliferative activity assessments. This study represents the first investigation of PA profiles in *F. fomentarius*, with total PA levels ranging from 32.67 to 2910.09 nmol/g dry weight (d.w.). The Bosnian strain exhibited the highest PA levels, with spermidine (SPD) concentrations ranging from 899.96 to 2910.09 nmol/g d.w. LC-MS/MS analysis identified several bioactive phenolics, including amentoflavone, baicalein, chrysoeriol, esculetin, and scopoletin—reported here for the first time in this fungus. The H_2_O and EtOH extracts from the Croatian and Serbian strains showed higher TPC levels, correlating with notable antioxidant activity. The EtOH and MeOH extracts demonstrated significant anti-acetylcholinesterase and antiproliferative activities, emphasizing their medicinal potential. These findings highlight the therapeutic potential of polar extracts from Balkan *F. fomentarius*.

## 1. Introduction

Mushrooms have long been valued for their medicinal properties due to the presence of bioactive compounds with significant health-promoting effects [1,2]. These properties include antidiabetic, anti-inflammatory, antiproliferative, antimicrobial, antioxidant, and neuroprotective activities [3,4,5,6,7,8,9]. Over 2000 species of edible and medicinal mushrooms have been documented, many of which have been used in traditional medicine across various regions, particularly in Asia. Key species such as *Fomes fomentarius*, *Cordyceps sinensis*, *Ganoderma lucidum*, *Lentinula edodes*, and *Lenzites betulinus* have long been integral to folk remedies in countries like China, Japan, Korea, and India for the treatment of various ailments, including inflammation, cancer, and neurodegenerative diseases [1,2,10,11,12,13].

Reactive oxygen species (ROS), known for their role in oxidative stress, have been linked to numerous diseases, including cancer and Alzheimer’s disease [14]. Alzheimer’s disease, characterized by a deficiency in acetylcholine, is often targeted using acetylcholinesterase (AChE) inhibitors. Mushrooms, owing to their notable antioxidant potential, offer a promising approach for managing oxidative stress and related pathological conditions [3]. While their anti-AChE and antioxidant properties have been largely attributed to polysaccharides and phenolic compounds, secondary metabolites like polyamines (PAs) remain underexplored [8]. PA degradation contributes to oxidative stress by generating reactive aldehydes and ROS, partly due to reduced PA levels. However, PAs themselves act as potent antioxidants and ROS scavengers [15]. The age-related decline in PA levels has long been associated with cognitive and behavioral impairments [16]. This observation supports the hypothesis that maintaining SPD levels during aging may promote longevity, potentially achievable through dietary supplementation to restore PA levels [17].

Among these, *F. fomentarius* (L.) Fr., known as “Iceman’s mushroom” due to its discovery alongside a 5000-year-old mummy, has demonstrated a range of therapeutic properties, including antioxidant, anti-inflammatory, anti-AChE, and antiproliferative effects [10,18,19].

*F. fomentarius* is widely distributed across the Northern Hemisphere, typically growing on hardwood trees such as beech, birch, and oak [12,20,21]. The medicinal potential of both its fruiting bodies and mycelia has been extensively studied, with recent research highlighting its rich mycochemical composition, including phenolics, benzofurans, coumarins, triterpenoids, and polysaccharides like β-glucans [10,12,16]. These compounds are linked to the mushroom’s antiproliferative and antioxidant activities, with specific polyphenols and triterpenoids playing crucial roles in modulating oxidative stress and inhibiting cell proliferation [11,22]. Furthermore, studies have shown that bioactive compounds in *F. fomentarius* can reduce AChE activity, suggesting its potential as a neuroprotective agent in conditions such as Alzheimer’s disease [3,23].

Ethnological studies reveal that *Fomes fomentarius* (tinder fungus or hoof fungus) has a longstanding role in traditional medicine and various practical applications. It is one of the earliest documented medicinal mushrooms, used for fire-starting, preservation, first aid, insect repellents, and spiritual rituals [10,24,25]. Fruiting bodies were employed in ceremonial smoking rituals in Germany and Austria and in West Siberia, where the Khanty burned them to ward off the deceased’s influence [10].

*F. fomentarius* was used to treat bladder disorders, dysmenorrhea, hemorrhoids, gastroenteric issues, inflammation, pain, and various cancers [10,22,25]. It featured in a Japanese anticancer beverage [18] and is noted in the traditional pharmacopeias of China, India, Korea, and Hungary for addressing cirrhosis, gastrointestinal disorders, inflammation, and oral ulcers [12,24]. Referred to as “mykes” in ancient Greece, it first appeared during Hippocrates’ era for cauterizing wounds and treating inflamed organs [24].

The Okanagan-Colville Indians used it for rheumatism, and it was widely applied as a styptic, earning the name “agaric of the surgeons” [25]. Hieronymus Bock recommended it as an emetic against mushroom poisoning in the 15th century. Moreover, it was used globally as an iodine-infused dressing for wounds and burns and sold in pharmacies as styptic bandages [10,25].

Despite its extensive global use, data on traditional applications of *F. fomentarius* in the Balkan region remain scarce. One study documented aspects such as harvesting methods, timing, storage techniques, preparations, and the ethnomycological use of its fruiting bodies [20]. Unique preparations of this species were traditionally prevalent in Corund, Lupeni, Odorheiu Secuiesc, and Sumuleu Ciuc in Transylvania (Romania). In other areas of the Balkan region, there is a lack of data indicating the use of this species for pharmacological purposes, although it was noted that people in certain regions, such as Croatia and Bulgaria, were aware of its health benefits [26,27]. Alongside very limited information regarding the ethnomedicinal use of this species in Serbia, Živković et al. [28] conducted the first ethnomycological study, documenting the use of 85 fungal species from 28 families for food and medicinal purposes, among which *F. fomentarius* (known as “trud” in Serbian) was used preventatively in the form of tea (one spoon of dried and ground mushroom is boiled in 1 L of water for 30 min). Collectively, this suggests a mycophilic consumption behavior among people in the Balkan region, excluding Slovenia [29].

In this study, we conducted a comparative analysis of three strains of *F. fomentarius* collected from the Balkan region, focusing on their mycochemical composition and biological activities. This research aims to provide a comprehensive understanding of the chemical diversity and biological potential of these unexplored strains, which could offer significant benefits in the prevention and treatment of oxidative stress-related diseases such as cancer and neurodegenerative disorders.

## 2. Materials and Methods

### 2.1. Mushroom Material

**Figure 1:**
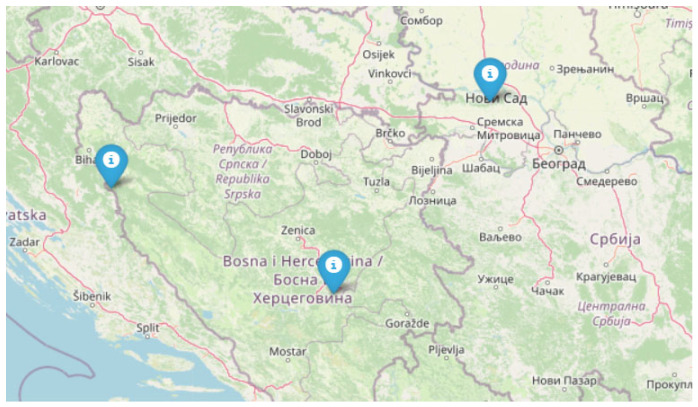
Geographical distribution of sampled *Fomes fomentarius* specimens across the Balkans. The map shows sampling localities in Croatia (FC), Bosnia and Herzegovina (FB), and Serbia (FS). The designated markers indicate specific collection sites: “Štrbački Buk” National Park (FC), Vrelo Bosne (FB), and “Fruška Gora” National Park (FS). (Map image sourced from Google Maps).

Fruiting bodies of *F. fomentarius* (Ph. Basidiomycota, Cl. Agaricomycetes, O. Polyporales, Fam. Polyporaceae, Gen. *Fomes*) were collected in January 2018 from “Štrbački Buk” National Park, Donji Lapac, Croatia (FC; 44.5594° N, 16.0033° E); in April 2018 from “Fruška Gora” National Park, Serbia (FS; 45.1678° N, 19.7140° E); and in October 2018 from Vrelo Bosne, Sarajevo, Bosnia and Herzegovina (FB; 43.8161° N, 18.2714° E) (Figure 1).

The identification of mushroom species was based on detailed macroscopic (color, shape, size and hyphal structures) and microscopic morphological characteristics, using specific identification keys as described in Rašeta et al. [6]. The samples were identified and authenticated by Dr. Eleonora Čapelja and subsequently deposited in the ProFungi Laboratory, Department of Biology and Ecology, Faculty of Sciences, University of Novi Sad, under the following voucher numbers: FC 12-00725, FS 12-00726, and FB 12-00662. Although molecular identification such as ITS (rDNA) barcoding was not performed due to the unavailability of suitable material for DNA extraction (as the fruiting bodies were fully processed for chemical and biological analyses), species determination was conducted with great care. Multiple authoritative taxonomic keys were consulted, and identification was confirmed by experienced mycologists. We acknowledge this as a limitation of the study and intend to incorporate molecular confirmation in future work to enhance taxonomic accuracy and reproducibility.

### 2.2. Extract Preparation

The fruiting bodies were sectioned, air-dried, and stored in dark containers at room temperature. The detailed procedure for extract preparation can be found in Rašeta et al. [30], where hot water (H_2_O), hydroethanolic (EtOH), chloroform (CHCl_3_), and hydromethanolic (MeOH) extracts were prepared according to the methods outlined by Rašeta et al. [4] and Gąsecka et al. [31]. The powdered fruiting bodies were mixed with solvent at a ratio of 1:10. Samples were macerated (IKA KS 4000i control) for 8 h, centrifuged (Sigma 3–30 K, Burlington, MA, USA) at 3999× *g* for 15 min at 4 °C, filtered through Whatman No.4 paper (Maidstone, Kent, UK), and evaporated under vacuum (Büchi R-210; Buchi Labortechnik AG, Flawil, Switzerland) at 40 °C to dryness, except for the H_2_O extract, which was lyophilized (ChristAlpha, Martin Christ Gefriertrocknungsanlagen GmbH, Osterode am Harz, Germany). The dry residues were dissolved in dimethyl sulfoxide (DMSO). All prepared extracts were stored at 4 °C for further use.

### 2.3. Mycochemical Characterization

#### 2.3.1. Atomic Absorption Spectrophotometry (AAS)

Macro- (Ca and Mg) and microelements (Cr, Cu, Mn, Fe, and Zn) in the powdered fungal samples were quantified using atomic absorption spectrophotometry (FS AAS240/GTA120, Agilent) following the method described by Kebert et al. [32].

#### 2.3.2. HPLC-FD Determination of Selected Polyamine Content

The PA profile and content of three main PAs, putrescine (PUT), SPD, and spermine (SPM), were determined in the mushroom material using high-performance liquid chromatography coupled with a fluorescence detector (HPLC-FD) using a method described by Scaramagli et al. [33], after derivatization of PAs with dansyl-chloride as a pre-treatment.

#### 2.3.3. LC/MS-MS Quantification of Phenolic Compounds and Total Phenolic Content (TPC) Determination

To quantify the selected phenolic compounds and quinic acid in the analyzed extracts, we utilized the liquid chromatography coupled with tandem mass-spectrometric detection (LC-MS/MS) technique in accordance with the protocol established by Orčić et al. [34]. The concentrations of standard phenolic compounds and quinic acid were determined by referencing a calibration curve generated from a series of dilutions of a standard mixture. Data were collected in dynamic MRM mode, with peak areas quantified using MassHunter software. Calibration curves were generated using OriginPro. Detailed retention times and MS parameters are provided in Table S1.

The total phenolic content (TPC) was measured using the Folin–Ciocalteu (FC) method, as described by Singleton et al. [35]. This approach relies on the spectrophotometric detection of phenolic compounds through their reaction with FC reagent, forming a colored complex. Absorbance was measured at 760 nm, and TPC was expressed as milligrams of gallic acid equivalents per gram of dry weight (mg GAE/g d.w.), based on a standard calibration curve.

#### 2.3.4. Determination of Total Carbohydrate Content

The total carbohydrate content (TCC) was determined using the phenol–sulfuric acid method, following the procedure outlined by Rašeta et al. [6]. This method involved the use of concentrated H_2_SO_4_ and 5% phenol. Absorbance was recorded at 490 nm for glucose, and a calibration curve was generated to quantify TC in the fungal extracts. The results were expressed as milligrams of glucose equivalents per gram of dry weight (mg GluE/g d.w.).

### 2.4. Biological Activities

#### 2.4.1. Determination of Antioxidant Activity

Antioxidant activity was evaluated through a series of standard radical scavenging assays, including DPPH [36], ABTS [37], and NO radical scavenging [38]. Additionally, Fe^2+^/ascorbate-induced lipid peroxidation was assessed [39], and the reducing power of the fungal extracts was measured using the FRAP assay [40].

In the DPPH assay, a mixture of 60 μL of 90 µM DPPH reagent, 180 μL of MeOH, and 10 μL of extract/standard was incubated in the dark at room temperature for 30 min, and the absorbance was measured at 515 nm [36]. For the ABTS assay, 290 μL of ABTS reagent (7 mM ABTS, 2.45 mM K_2_S_2_O_8_) was mixed with 10 μL of extract/standard, incubated at room temperature for 5 min, and the absorbance was recorded at 734 nm [37]. The NO radical scavenging assay involved mixing 15 μL of extract/standard, 250 μL of sodium nitroprusside (10 mmol/L), and 250 μL of phosphate buffer (pH 7.4). After 90 min of light exposure, 500 μL of Griess reagent was added, and the absorbance was measured at 546 nm [38]. Lipid peroxidation inhibition was assessed using a TBA assay with polyunsaturated fatty acids derived from linseed oil (69.7% linolenic acid, 13.5% linoleic acid). The fatty acids were emulsified in phosphate buffer containing 0.25% Tween-80, sonicated for 1 h, and mixed with FeSO_4_, ascorbic acid, and test samples (0.13–232 mg/mL). After a 1 h incubation at 37 °C, EDTA and a TBA mixture were added, followed by heating, centrifugation, and absorbance measurement of malondialdehyde (MDA) at 532 nm [39]. The FRAP assay involved adding 10 μL of extract/standard to 225 μL of FRAP reagent (TPTZ, FeCl_3_, acetate buffer) and 22.5 μL of distilled water (dH_2_O), with the absorbance recorded at 593 nm after 6 min [40].

The radical scavenging capacity (RSC) for DPPH and lipid peroxidation inhibition is expressed as IC_50_ values (μg/mL), whereas for NO radicals, RSC is expressed as IC_25_ values (μg/mL). For the ABTS assay, the results were derived from a Trolox calibration curve and expressed as milligrams of Trolox equivalents per gram of dry weight (mg TE/g d.w.). For the FRAP assay, the reduction potential was calibrated using ascorbic acid, with the results expressed as milligrams of ascorbic acid equivalents per gram of dry weight (mg AAE/g d.w.). Propyl gallate (PG) was used as the positive control in all assays.

#### 2.4.2. Determination of Anti-Acetylcholinesterase Activity (Anti-AChE)

To assess the neuroprotective activity of the fungal extracts, a modified method by Ellman et al. [41], as described in Pintać et al. [42], was used. Galantamine served as the positive control for comparison of its IC_50_ value with that of the extracts.

#### 2.4.3. Determination of Antiproliferative Activity

Cell culturing

Human breast cancer cell lines (MCF7, T47D, and MDA-MB-231), a cervical cancer cell line (HeLa), and an ovarian cancer cell line (A2780) were purchased from ECACC (European Collection of Cell Cultures, Salisbury, UK), while SiHa cells (cervical cancer) were obtained from ATCC (American Tissue Culture Collection, Manassas, VA, USA). The cells were cultivated in a minimal essential medium supplemented with 10% fetal bovine serum, 1% nonessential amino acids, and an antibiotic-antimycotic mixture. All media and supplements were purchased from Lonza Group Ltd. (Basel, Switzerland). The cells were cultivated at 37 °C in a humidified atmosphere containing 5% CO_2_.

Antiproliferative activity (MTT assay)

The growth inhibitory activity of the extracts was determined using the MTT method against a panel of human cancer cell lines of gynecological origin [43].

### 2.5. Statistical Analysis

Three different strains of *F. fomentarius* were utilized and each experiment was conducted three times, except for the examination of antiproliferative activity, which was performed twice. The outcomes were reported as average values with either standard deviation (SD) or standard error of the mean (SEM), depending on the antiproliferative assay. One-way analysis of variance (ANOVA) and Tukey’s HSD test were carried out to test for any significant differences between the means; the mean values of antioxidant activities between two extracts or two treatments were analyzed by an independent samples t-test. Correlations were obtained with Pearson’s correlation coefficient in bivariate correlations. Differences between means at a 5% (*p* < 0.05) level were considered significant. For defining the variability in chemical composition and biological activity between the investigated extracts, principal component analysis (PCA) was applied using PAST version 3.16 [44].

## 3. Results and Discussion

### 3.1. Mycochemical Profile

#### 3.1.1. Macro- and Microelement Content and Biological Significance

**Figure 2:**
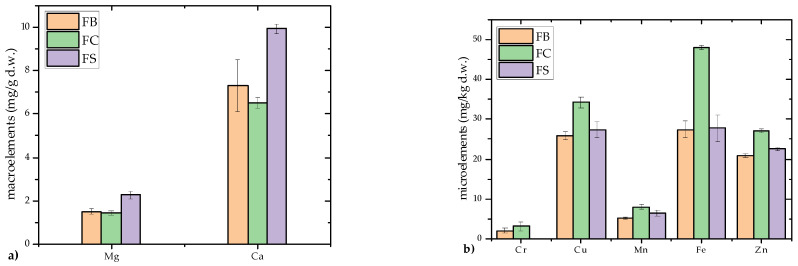
Mineral composition of dried samples of *Fomes fomentarius* from three different Balkan localities. (**a**) Macroelement content (mg/g d.w.); (**b**) Microelement content (mg/kg d.w.). Strains are labeled as follows: FB—Bosnia and Herzegovina; FC—Croatia; FS—Serbia. Data are presented as mean ± standard deviation (SD). Statistical analysis was performed using one-way ANOVA followed by Tukey’s Honestly Significant Difference (HSD) post hoc test (*p* < 0.05). Abbreviation: d.w.—dry weight.

The mineral composition of *F. fomentarius* included two macroelements (Mg and Ca) and five microelements (Cr, Cu, Mn, Fe and Zn), as shown in Figure 2a,b.

Among the analyzed strains, the Serbian strain (FS) exhibited the highest mean concentrations of macroelements, with Ca at 9.93 ± 0.21 mg/g d.w. and Mg at 2.28 ± 0.17 mg/g d.w. Across all strains, Ca was found in significantly higher concentrations (7.30–9.93 mg/g d.w.) compared to Mg (1.42–2.28 mg/g d.w.), resulting in relatively high Ca/Mg ratios. This Ca/Mg ratio is biologically significant due to its critical role in physiological processes, particularly bone health [45]. An optimal balance of calcium and magnesium is essential for calcium absorption, bone mineralization, and the prevention of conditions such as osteoporosis [46]. Low magnesium intake can impair calcium metabolism and decrease bone density, while a favorable Ca/Mg ratio has been linked to improved skeletal outcomes in both animal and human studies [47]. Therefore, *F. fomentarius*—particularly the FS strain—may represent a valuable dietary source of calcium with a beneficial Ca/Mg profile. The observed high calcium content likely reflects the mineral-rich wood substrates of this parasitic/saprotrophic species. Similar patterns were reported by Karaman and Matavulj [48], who found elevated Ca concentrations in lignicolous mushroom substrates compared to wild-growing fungi.

Regarding microelements, significant accumulation of Zn, Fe, and Cu was noted, while Mn and Cr were present in lower concentrations (Mn: 5.24–8.05 mg/kg d.w.; Cr: 2.05–3.12 mg/kg d.w., *p* < 0.01). Fe was the most abundant, with the Croatian strain (FC) showing the highest Fe concentration (48.07 ± 0.54 mg/kg d.w.), followed by the FB and FS strains (27.36–27.78 mg/kg d.w.). This aligns with previous findings of Fe hyperaccumulation in lignicolous fungi such as *Meripilus giganteus* and *F. fomentarius* [48]. Cu was the second most prevalent element (25.90–34.27 mg/kg d.w.), with Zn also present at substantial levels, both essential for human health [49]. The FC strain had the highest Cu and Zn contents, consistent with earlier studies on Cu and Zn accumulation in lignicolous and tericolous fungi [48]. Mn and Cr were detected in lower amounts, ranging from 5.24 to 8.05 mg/kg d.w. for Mn and 2.05 to 3.12 mg/kg d.w. for Cr (*p* < 0.01). These concentrations are comparable to previously published data on edible fungi from Serbia [48] and India [49].

Compared to Turkish strains [50], Balkan *F. fomentarius* exhibited significantly higher concentrations of Ca and Mg, reinforcing its value as a natural source of minerals vital for bone health and osteoporosis prevention [51]. Notably, Fe levels approached recommended daily intake values, suggesting potential applications as a dietary supplement [49].

Although Serbian researchers have extensively reported on the mineral composition of various lignicolous and tericolous mushrooms [4,48,52,53,54], this study presents the first comparative analysis of *F. fomentarius* strains from the Balkan region. These results underscore the species’ capacity to effectively accumulate essential biogenic minerals, particularly Fe, and reveal notable inter-strain differences likely influenced by substrate type and geographic origin.

Environmental factors, including habitat, geographical location, and substrate type, significantly affect mineral uptake [4,48]. Additional influences include genotype, ecotype, growth conditions, and mycelial age [49,55]. For example, strains from the Balkans exhibited much higher macroelement concentrations than those from Uşak, Turkey, where Ca and Mg levels were reported at 0.1814 mg/g d.w. and 0.1816 mg/g d.w., respectively [50].

These findings emphasize the adaptability of *F. fomentarius* in mineral absorption directly from the wood substrate and indirectly from the surrounding soil, supporting its reputation as one of the most effective accumulators of Fe among fungi [48,56]. The high mineral content of *F. fomentarius* further validates its potential as a source of bioactive compounds and food supplements, particularly for mitigating mineral deficiencies and promoting bone health. However, its hard texture makes it unsuitable for direct consumption, necessitating its use in processed forms such as extracts or supplements [25].

#### 3.1.2. Content of Polyamines 

Despite extensive research on PAs in plant- and animal-derived foods, their occurrence in wild-growing mushrooms remains notably scarce [57]. In mushrooms, SPD is generally the dominant PA, while putrescine (PUT) and SPD are associated with a range of morphological, physiological, and metabolic processes. SPM, however, is not consistently present in mushrooms [15].

**Figure 3:**
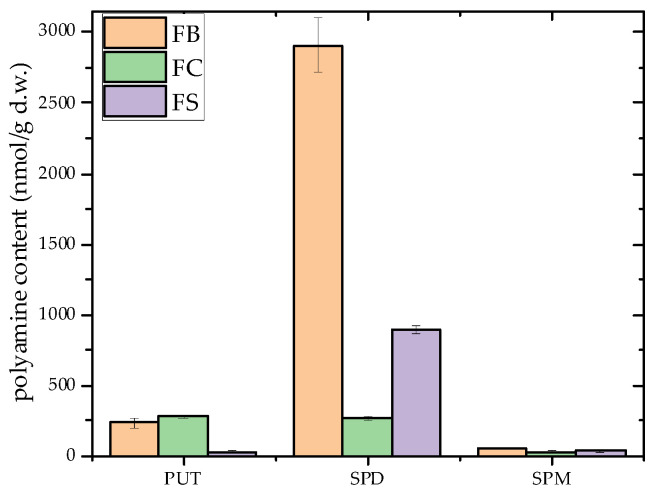
Polyamine content of dried samples of *Fomes fomentarius* from three different Balkan localities, expressed as putrescine, spermidine, and spermine levels (nmol/g d.w.). Strains are labeled as follows: FB—Bosnia and Herzegovina; FC—Croatia; FS—Serbia. Data are presented as mean ± standard deviation (SD). Statistical analysis was performed using one-way ANOVA followed by Tukey’s Honestly Significant Difference (HSD) post hoc test (*p* < 0.05). Abbreviation: d.w.—dry weight.

This study is the first to investigate the PA profile in *F. fomentarius,* analyzing three strains collected from the Balkan region. The results confirmed the presence of all three PAs, with total levels ranging from 32.67 to 2910.09 nmol/g d.w. (Figure 3). Among the strains, the FB strain exhibited the highest concentrations, while the FC strain had the lowest.

SPD was found to be the predominant PA across the strains, with concentrations ranging from 899.96 to 2910.09 nmol/g d.w., except in the FC strain, where its concentration was significantly lower (270.65 ± 14.72 nmol/g d.w.). The FB strain had the highest SPD levels (2910.09 ± 196.45 nmol/g d.w.). These findings indicate that SPD may be commonly present in *F. fomentarius*, although its concentration varied substantially among the analyzed strains. SPM was present in the lowest concentrations (32.67–54.35 nmol/g d.w.), with the FB strain again showing the highest level (54.35 ± 3.02 nmol/g d.w.). By contrast, PUT was predominant in the FC strain (278.56 ± 5.58 nmol/g d.w.), with concentrations higher than those reported for some edible wild mushrooms [57,58].

The results align with the observations by Dadáková et al. [57], who noted significantly higher levels of SPD in fungi compared to other foods. The SPD concentrations in *F. fomentarius* determined in this study far exceed those reported for other fungal species, such as *A. bisporus*, *L. edodes*, and *Pleurotus* spp., which range between 32.30–71.30 mg/kg d.w. [58], and *A. bisporus* and *X. badius*, with values of 404–604 mg/kg d.w [57]. SPD plays a crucial role in cellular metabolism and growth, and its abundance in fungi has been previously documented as among the highest found in food [57,59].

The remarkably high SPD levels detected in *F. fomentarius* may be attributed to several species-specific physiological and ecological factors. *F. fomentarius* is a long-lived perennial polypore with robust wood-decaying capabilities and the ability to form dense, persistent fruiting bodies that can remain viable over extended periods [12]. Unlike the short-lived fruiting bodies of species such as *A. bisporus* or *Pleurotus* spp., which are typically ephemeral [9], the perennial and woody structures of *F. fomentarius* may enable cumulative polyamine accumulation over time. This extended lifespan allows for prolonged metabolic activity, potentially contributing to the gradual buildup of polyamines such as SPD. While specific longitudinal studies of polyamine accumulation in *F. fomentarius* are lacking, research on other long-lived fungi has demonstrated significant levels of these compounds [8], suggesting that a similar trend may apply. Moreover, polyamines like SPD are involved in stress tolerance, maintenance of cell wall integrity, and fungal morphogenesis [15]—all of which are critical for ligninolytic fungi that colonize and decompose resilient, nutrient-poor woody substrates [16]. Elevated SPD levels may thus reflect an adaptive response to persistent environmental stress and interspecific competition within the wood decay niche. Previous studies have also linked high polyamine concentrations to oxidative stress responses and sustained substrate colonization, further supporting this hypothesis. Nonetheless, additional research is needed to elucidate the regulatory mechanisms governing polyamine biosynthesis and storage in this ecologically and biochemically distinct species.

To our knowledge, the SPD levels detected in this study represent the highest recorded in mushrooms to date. Based on these findings, *F. fomentarius* may be considered a promising and underexplored source of PAs, particularly SPD. However, given the limited number of strains analyzed, further investigation involving a broader population of *F. fomentarius* is required to confirm ubiquity and variability of SPD content in this species. According to the classification proposed by Kalač [59], the analyzed strains constitute a significant reservoir of bioactive PAs. Nevertheless, additional studies are needed to fully elucidate the biological roles of these compounds and to assess their potential applications as dietary supplements or therapeutic agents.

#### 3.1.3. Phenolic Content Determined by LC-MS/MS

The LC-MS/MS analysis of the *F. fomentarius* extracts revealed quinic acid (58.99–996.78 μg/g d.w.) and scopoletin (23.63–853.07 μg/g d.w.) as the predominant compounds, particularly in the FS and FB strains (Table 1 and Figure S1).

These compounds were identified in *F. fomentarius* for the first time. The highest concentrations were found in the FB strain’s MeOH extract (quinic acid, 996.78 ± 99.68 μg/g d.w.) and the FS strain’s EtOH extract (scopoletin, 853.07 ± 68.25 μg/g d.w.). Quinic acid was detected in almost all extracts, except for the nonpolar CHCl_3_ extracts and the FS EtOH extract (Figure S1). Additionally, chlorogenic acid, a derivative of quinic acid, was identified at 6.74 ± 0.34 μg/g d.w. in the MeOH extract, although its concentration was much lower than previously reported by Bal et al. [60].

Among the flavonoids, baicalein was detected only in the FS strain, specifically in the EtOH and H_2_O extracts (44.63 ± 8.93 μg/g d.w. and 147.50 ± 29.50 μg/g d.w., respectively). Amentoflavone was also identified, marking its first occurrence in *F. fomentarius*. The FC strain’s CHCl_3_ extract was notable for having the highest concentration of baicalein and the exclusive presence of amentoflavone, indicating a unique phenolic profile.

**Figure 4:**
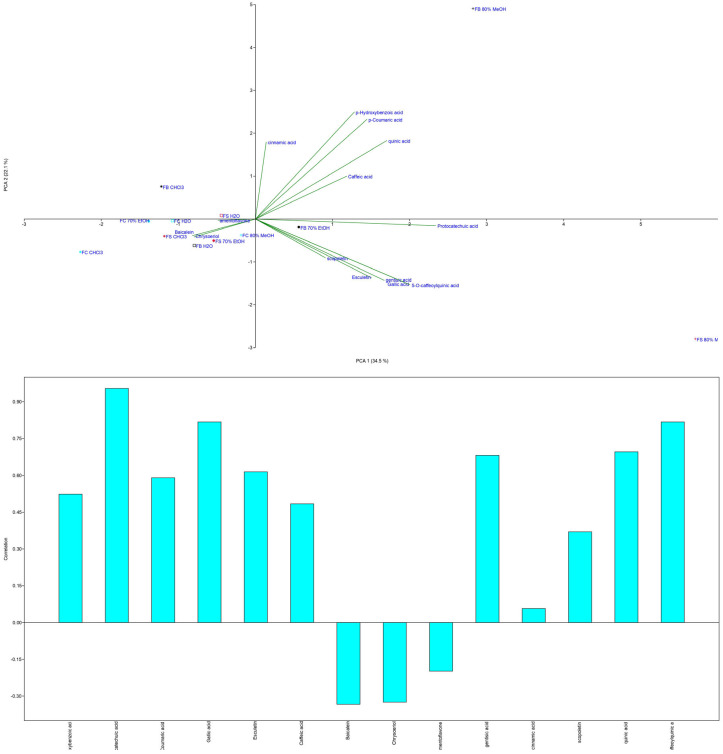
PCA analysis of *Fomes fomentarius* extracts and identified polyphenolics using LC-MS/MS analysis. Distribution of variables in (above) the score plot and (below) the loading plot of the first two principal components. The following abbreviations are used for the examined parameters: FB—strain sampled from Bosnia and Herzegovina; FC—strain sampled from Croatia; FS—strain sampled from Serbia; 70% EtOH—hydroethanolic extract prepared with 70% EtOH; H_2_O—hot water extract; 80% MeOH—hydromethanolic extract prepared with 80% MeOH; CHCl_3_—chloroform extract.

To identify strain-specific compounds and evaluate the impact of solvents, principal component analysis (PCA) was applied (Figure 4).

PCA revealed variances of 34.5% for PC1 and 22.1% for PC2. Baicalein significantly contributed to the negative part of PC1, while protocatechuic acid had the greatest loading in the positive part. The FC strain clustered uniquely in the negative part of both axes, with the highest baicalein content and exclusive presence of chrysoeriol and amentoflavone. By contrast, the FB MeOH extract, which was located in the positive parts of both axes, had the highest concentrations of all tested phenolic compounds, including quinic acid.

Hydroxycinnamic acids were more prevalent than hydroxybenzoic acids in the extracts, often found as glycosylated derivatives or esters of quinic acid, shikimic acid, or tartaric acid [61]. The presence of quinic acid and scopoletin in *F. fomentarius* was notable, as quinic acid had not been detected in previous studies [62]. Compared to other mushrooms, such as *Coprinus comatus* and *C. truncorum* from Serbia [63], *F. fomentarius* contained significantly higher quinic acid levels, ranging from 1.59 to 215 μg/g d.w. Similarly, scopoletin concentrations in *F. fomentarius* were much higher than those reported for *C. comatus* and *C. truncorum* (1.11–1.97 μg/g d.w.) in previous studies using the same method [34]. Regarding the most present compounds, quinic acid (996.78 μg/g d.w. in the MeOH extract; 414.18 μg/g d.w. in the EtOH extract) and scopoletin (853.07 μg/g d.w. in the MeOH extract) were the predominant phenolic compounds identified in *F. fomentarius*. While quinic acid is a common metabolite in fungi, its concentration in *F. fomentarius* notably exceeds levels typically reported for other medicinal polypores, such as *Ganoderma* spp. (3.05–10.90 μg/g d.w.) [6], *Trametes versicolor* (~200–400 μg/g d.w.) [64], and *Stereum subtomentosum* (9.70–191.60 μg/g d.w.) [65]. These results suggest that *F. fomentarius* is a particularly rich source of this compound, which is known for its broad-spectrum bioactivities, including antioxidant, antimicrobial, and antidiabetic effects [66]. Scopoletin, by contrast, is a coumarin compound rarely detected in fungi. To our knowledge, this is the first report of scopoletin in *F. fomentarius*, positioning it as a novel source. Scopoletin has demonstrated notable antifungal properties, including 68.2% inhibition of *Candida tropicalis* biofilms at its MIC, which is significantly more effective than fluconazole (250 μg/L vs. 50 μg/L MIC) [67]. Han et al. [68], summarized that scopoletin can activate some key antioxidant enzymes, such as superoxide dismutase (SOD), glutathione peroxidase (GPX), and glutathione-*S*-transferase (GST), thereby enhancing the cellular antioxidant defense system. The presence of scopoletin has also been confirmed in the wild edible fungi *Chroogomphus rutilus*.

The unusually high levels observed in *F. fomentarius* are thus ecologically and pharmacologically relevant. The co-occurrence of quinic acid and scopoletin in substantial concentrations may contribute to synergistic antioxidant and antifungal effects, offering a promising profile for potential therapeutic applications. Further studies are warranted to explore this synergistic effect and to elucidate the underlying biosynthetic pathways.

A total of six novel phenolic compounds were identified in *F. fomentarius* extracts—amentoflavone, baicalein, chrysoeriol, cinnamic acid, esculetin, and scopoletin—along with quinic acid, bringing the total number of phenolic compounds reported for this species to 19, as documented in various studies [23,60,62,69]. Among them, chrysoeriol and cinnamic acid were identified, with concentrations below the limit of quantification (LoQ) but above the limit of detection (LoD).

Comparative analyses indicated that the Serbian strain exhibited a greater diversity and quantity of phenolics compared to strains from Poland [69] and Turkey [23,60].

The phenolic content was highest in the FB and FS strains, while the FC strain exhibited relatively lower phenolic levels. This disparity could be attributed to the presence of other bioactive or unexamined phenolics in the FC strain, along with factors such as geographical origin, climate differences, sampling periods, and the age of the fruiting bodies. For example, the FC strain was collected during the winter, which may have influenced its phenolic profile.

Consistent with prior research [4,62,63], the highest phenolic content was typically extracted using polar solvents such as MeOH and EtOH. These results emphasize that the phenolic profile of *F. fomentarius* is strongly influenced by both the strain and the extraction solvent used.

#### 3.1.4. TPC and TCC Content

**Figure 5:**
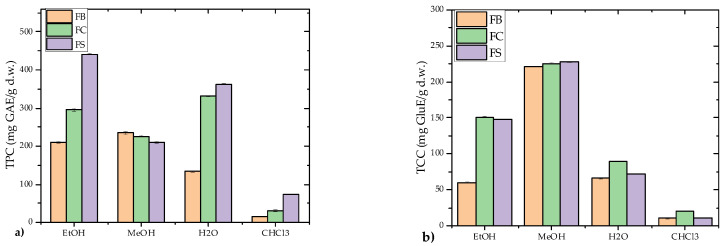
Total phenolic content (TPC) and total carbohydrate content (TCC) of *Fomes fomentarius* extracts from three different Balkan localities. (**a**) Total phenolic content (TPC); (**b**) Total carbohydrate content (TCC). Strains are labeled as follows: FB—Bosnia and Herzegovina; FC—Croatia; FS—Serbia. Extracts include: EtOH—70% hydroethanolic extract; MeOH—80% hydromethanolic extract; H_2_O—hot water extract; CHCl_3_—chloroform extract. Results are expressed as gallic acid equivalents (GAE) for TPC and glucose equivalents (GluE) for TCC, both per gram of dry weight (d.w.). Data are presented as mean ± standard deviation (SD). Statistical analysis was performed using one-way ANOVA followed by Tukey’s Honestly Significant Difference (HSD) post hoc test (*p* < 0.05).

The total phenolic content (TPC) and total carbohydrate content (TCC) of *F. fomentarius* strains were evaluated across various extracts (Figure 5a,b).

Among the extracts, the highest TPC was observed in the FS strain’s EtOH extract, with a value of 441.16 ± 1.85 mg GAE/g d.w., while the CHCl_3_ extracts exhibited the lowest TPC values, ranging from 14.30 to 72.55 mg GAE/g d.w. Similarly, the highest TCC was found in the FS strain’s MeOH extract, with a concentration of 227.20 ± 0.12 mg GluE/g d.w., while the lowest values were recorded in the CHCl_3_ extracts (10.24–20.26 mg GluE/g d.w.). These results highlight the efficiency of polar solvents (EtOH and MeOH) in extracting phenolic compounds and carbohydrates, consistent with prior findings that nonpolar extracts, primarily composed of triterpenoids and other nonpolar compounds, contain lower levels of phenolics and carbohydrates.

When compared to the values reported in the existing literature, the TPC values observed in this study were significantly higher. For example, Karaman et al. [62] reported TPC values of 43.06 ± 0.19 mg GAE/g d.w. and 82.54 ± 0.12 mg GAE/g d.w. for Serbian *F. fomentarius* samples prepared using EtOH and MeOH extracts [62], respectively, while the current study recorded considerably higher amounts in the polar extracts. Similarly, Orhan and Üstün [3] reported a TPC value of 47.29 ± 1.37 mg GAE/mg extract for 85% EtOH extracts of *F. fomentarius*. This study’s findings align with Karadeniz et al. [70], who noted that EtOH is highly effective in phenolic extraction. However, disparities in TPC among studies may be attributed to various factors, including geographical origin, climatic conditions, harvesting location, extraction methods, mushroom developmental stage, and whether results are expressed on a dry or fresh weight basis [69].

Regarding TCC, limited data are available for *F. fomentarius*. The TCC values observed in this study were lower than those reported in previous studies. Vazirian et al. [18] recorded a total polysaccharide content of 533.0 ± 2.0 mg/g, while Deveci et al. [71] reported a total carbohydrate content of 73.31 ± 6.35%. Despite these discrepancies, the MeOH extracts in this study consistently exhibited the highest carbohydrate concentrations. The variability in TCC can be influenced by environmental factors such as geographical origin, climate, and the developmental stage of fruiting bodies [72]. Additionally, the distribution of certain mushroom species is region-specific, influenced by climate, soil composition, rainfall patterns, and seasonal variations. These environmental factors affect their growth, ultimately influencing both their fruiting bodies and mycelial development [73].

The results highlight the superior efficiency of polar solvents (MeOH and EtOH) in extracting both TPC and TCC, corroborating prior research [4,62,63]. This underscores the influence of solvent polarity on phenolic and carbohydrate extraction from *F. fomentarius*. Furthermore, the notable differences in TPC and TCC across strains and extraction methods suggest that geographic origin and substrate composition significantly impact the composition of phenolics and carbohydrates in *F. fomentarius*.

### 3.2. Antioxidant Activity

**Figure 6:**
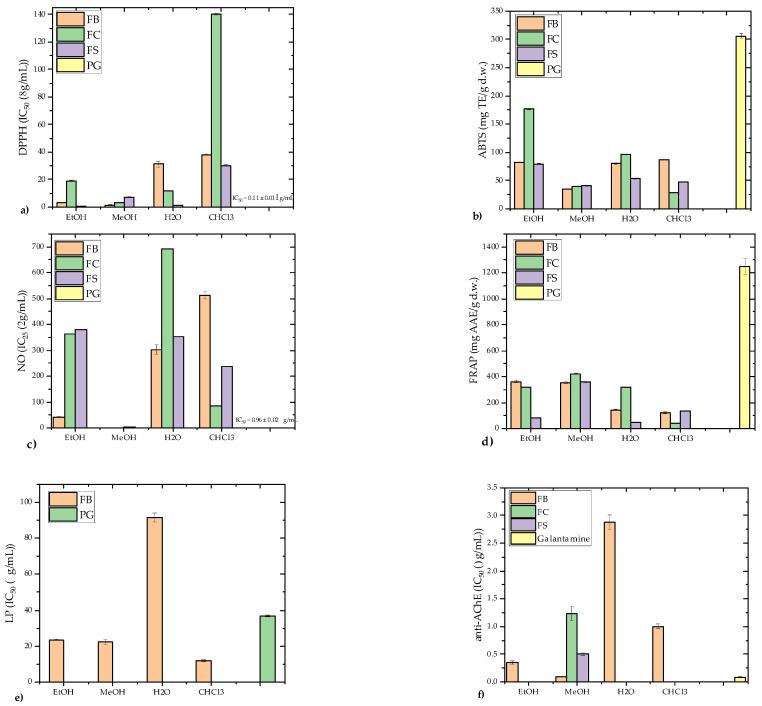
Antioxidant and anti-acetycholinesterase activities of *Fomes fomentarius* extracts from three Balkan localities. (**a**) DPPH scavenging activity; (**b**) ABTS scavenging activity; (**c**) NO scavenging activity; (**d**) Ferric reducing antioxidant power (FRAP assay); (**e**) Fe^2^⁺/ascorbate-induced lipid peroxidation (LP, tested only in FB strain extracts); (**f**) Anti-acetylcholinesterase (AChE) activity (tested only in FB strain extracts, and MeOH extracts of FC and FS). Strains are labeled as follows: FB—Bosnia and Herzegovina; FC—Croatia; FS—Serbia. Abbreviations: EtOH—hydroethanolic extract prepared with 70% EtOH; MeOH—hydromethanolic extract prepared with 80% MeOH; H_2_O—hot water extract; CHCl_3_—chloroform extract; PG—propyl gallate; d.w.—dry weight; DPPH—radical scavenger capacity against 2,2-diphenyl-1-picrylhydrazyl radical, DPPH^•^; ABTS—radical scavenger capacity against 2,2′-azino-bis(3-ethylbenzothiazoline-6-sulfonic acid), ABTS^•+^; NO—radical scavenging capacity against NO^•^; FRAP—ferric reducing antioxidant power; AAE—ascorbic acid equivalents; TE—Trolox equivalents. Data are expressed per gram of dry weight (d.w.) and presented as mean ± standard deviation (SD). Statistical analysis was performed using one-way ANOVA followed by Tukey’s Honestly Significant Difference (HSD) post hoc test (*p* < 0.05).

The antioxidant activities of *F. fomentarius* extracts were evaluated using DPPH, ABTS, NO scavenging, lipid peroxidation (LP) inhibition, and FRAP assays (Figure 6a–e).

The results, compared to the standard antioxidant propyl gallate (PG), revealed that the polar extracts (EtOH, MeOH) demonstrated significantly higher antioxidant activity than the nonpolar CHCl_3_ extracts, with one notable exception: the FB strain’s CHCl_3_ extract showed the strongest LP inhibition activity (IC_50_ = 11.88 ± 0.50 μg/mL) (Figure 6e). The FS strain’s EtOH extract exhibited the strongest DPPH scavenging activity (IC_50_ = 0.78 ± 0.02 μg/mL) (Figure 6a), while the FC strain’s EtOH extract demonstrated remarkable ABTS scavenging capacity (176.46 ± 0.46 mg TE/g d.w.) (Figure 6b). The FB strain’s MeOH extract showed the highest NO radical scavenging activity (IC_25_ = 0.27 ± 0.01 μg/mL) (Figure 6c), surpassing the activity of PG (IC_25_ = 0.96 ± 0.02 μg/mL). The FRAP assay results highlighted the MeOH extracts as having the strongest reducing power (352.19–423.78 mg AAE/g d.w.), closely followed by the FB strain’s EtOH extract (362.76 mg AAE/g d.w.) (Figure 6d).

The DPPH scavenging activities of the polar extracts (IC_50_ values ranging from 0.78 to 31.40 μg/mL) were notably stronger than the values reported in previous studies. For instance, Karaman et al. [62] reported a slightly higher IC_50_ value for EtOH extracts (10.87 ± 0.11 μg/mL). Orhan and Üstün [3] recorded moderate DPPH inhibition (24.32–26.14%) in 85% EtOH extracts at higher concentrations (250–5000 μg/mL), while Ilyashenka [74] found considerably weaker activity in ethyl acetate (IC_50_ = 515.6 ± 100.5 μg/mL) and EtOH (IC_50_ = 751.2 ± 19.3 μg/mL) extracts. Similarly, the ABTS scavenging capacity of the FC strain’s EtOH extract (176.46 ± 0.46 mg TE/g d.w.) aligned with the findings by Darkal et al. [75], who reported excellent ABTS activity (IC_50_ = 0.005–0.04% w/v) in *F. fomentarius*. By contrast, melanins extracted from *F. fomentarius* exhibited weaker antioxidant activity [76].

In the FRAP assay, the MeOH extracts consistently exhibited the strongest reducing power, reflecting their robust electron-donating ability. These results corroborate findings by Darkal et al. [75], who demonstrated superior FRAP activity in milled *F. fomentarius* samples. Interestingly, the exceptional LP inhibition by the FB strain’s CHCl_3_ extract, typically uncharacteristic for nonpolar extracts, warrants further investigation.

**Figure 7:**
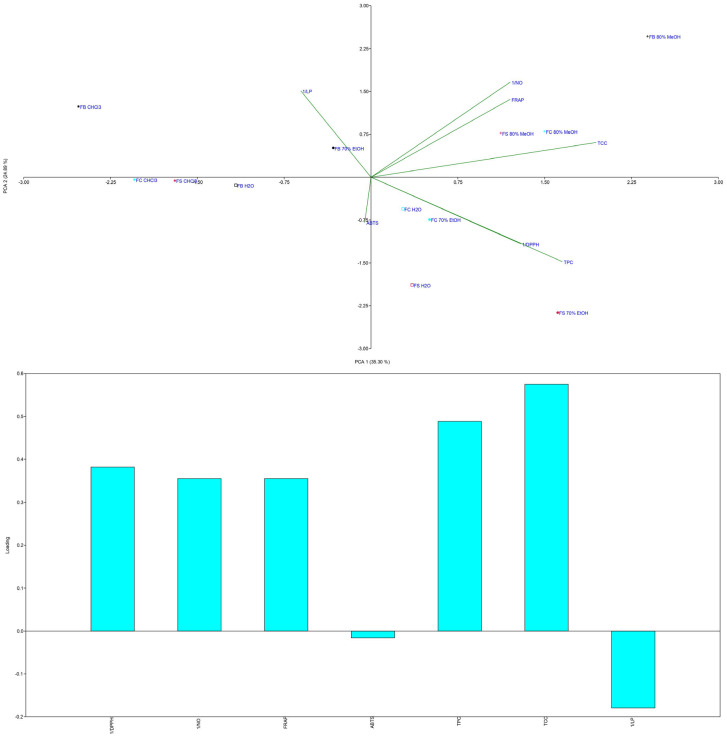
PCA analysis of analyzed *Fomes fomentarius* extracts and conducted antioxidant assays. Distribution of variables in (above) the score plot and (below) the loading plot of the first two principal components. The following abbreviations are used for the examined parameters: FB—strain sampled from Bosnia and Herzegovina; FC—strain sampled from Croatia; FS—strain sampled from Serbia; 70% EtOH—hydroethanolic extract prepared with 70% EtOH; H_2_O—hot water extract; 80% MeOH—hydromethanolic extract prepared with 80% MeOH; CHCl_3_—chloroform extract; DPPH–radical scavenger capacity against 2,2-diphenyl-1-picrylhydrazyl radical, DPPH^•^; ABTS—radical scavenger capacity against 2,2’-azino-bis(3-ethylbenzothiazoline-6-sulfonic acid), ABTS^•+^; NO—radical scavenging capacity against NO^•^; LP—lipid peroxidation assay; FRAP—ferric reducing antioxidant power.

**Figure 8:**
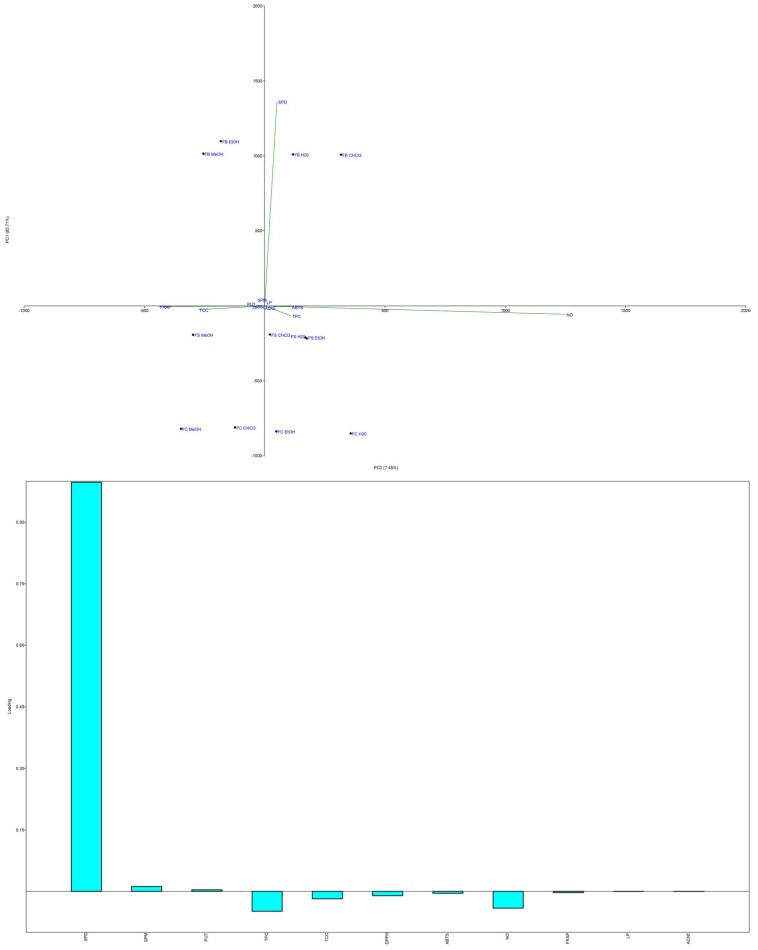
PCA analysis of analyzed *Fomes fomentarius* extracts, mycochemical profile including TPC, TCC, and PAs and conducted antioxidant assays and measured anti-AChE properties. Distribution of variables in (above) the score plot and (below) the loading plot of the first two principal components. The following abbreviations are used for the examined parameters: FB—strain sampled from Bosnia and Herzegovina; FC—strain sampled from Croatia; FS—strain sampled from Serbia; 70% EtOH—hydroethanolic extract prepared with 70% EtOH; H_2_O—hot water extract; 80% MeOH—hydromethanolic extract prepared with 80% MeOH; CHCl_3_—chloroform extract; anti-AChE—anti-acetylcholinesterase; DPPH—radical scavenger capacity against 2,2-diphenyl-1-picrylhydrazyl radical, DPPH^•^; ABTS—radical scavenger capacity against 2,2’-azino-bis(3-ethylbenzothiazoline-6-sulfonic acid), ABTS^•+^; NO—radical scavenging capacity against NO^•^; LP—lipid peroxidation assay; FRAP—ferric reducing antioxidant power; TPC—total phenolic content; TCC—total carbohydrate content; PAs—polyamines; PUT—putrescine; SPD—spermidine; SPM—spermine.

PCA of the antioxidant results (Figure 7 and Figure 8) revealed that the sample groups were influenced by the extraction solvents used.

The MeOH extracts from all three strains clustered in the first quadrant, indicating high NO scavenging and FRAP activity alongside the highest TCC levels. The CHCl_3_ extracts grouped in the negative part of PC1, showing the lowest antioxidant potential and TPC/TCC values. The FS and FC H_2_O and EtOH extracts grouped together, reflecting moderate antioxidant activity.

The results highlight a strong correlation between the antioxidant activity and the TPC and TCC levels of the extracts. This is consistent with previous research showing that the antioxidant potential of mushrooms is strongly associated with their phenolic content [4,5,61,62]. Phenolic compounds such as quinic acid and scopoletin play key roles in scavenging free radicals, with quinic acid known as a pro-metabolite of nicotinamide and tryptophan [77] and scopoletin acting as a superoxide scavenger [78].

In addition to phenolic compounds, carbohydrates, particularly polysaccharides, significantly contribute to the antioxidant potential of *F. fomentarius*. Polysaccharides are known to enhance the immune response and reduce oxidative stress through NO scavenging, as observed in samples with the highest TCC values [6]. Their antioxidant activity is influenced by factors such as monosaccharide composition, molecular weight, and protein content [79].

Interestingly, PAs, particularly SPD, may also contribute to antioxidant effects (Figure 7). The FB strain, with the highest PA content, exhibited notable NO scavenging activity. PAs act as free radical scavengers and metal chelators, mitigating oxidative damage, but can also generate pro-oxidative effects by producing hydrogen peroxide during catabolism [57,80]. This dual role underscores their complexity, as PAs may prevent chronic diseases while posing risks for cancer patients [59,81].

### 3.3. Anti-Acetylcholinesterase Activity

The neuroprotective potential of *F. fomentarius* extracts was evaluated by their potential to inhibit AChE, a critical therapeutic target in the treatment of Alzheimer’s disease and other neurodegenerative disorders. AChE inhibition enhances acetylcholine levels in the brain, improving cholinergic functions and alleviating Alzheimer’s disease symptoms [82]. FDA-approved AChE inhibitors such as galantamine and rivastigmine are primarily derived from natural sources [83,84,85], highlighting the importance of exploring bioactive compounds from fungi and other natural materials for therapeutic applications.

Among the analyzed extracts, the MeOH extract of the FB strain exhibited the strongest AChE inhibitory activity (IC_50_ = 0.099 mg/mL), followed by the EtOH (0.351 mg/mL) and CHCl_3_ (1.003 mg/mL) extracts, while the H_2_O extract had the weakest effect (IC_50_ = 2.881 mg/mL) (Figure 6f). These findings are consistent with prior studies showing weaker AChE inhibition in nonpolar extracts [8]. The potent activity of the FB strain extracts strongly correlated with their higher levels of PAs, particularly SPD and SPM. PAs have been linked to neuroprotective effects, memory enhancement, and the regulation of acetylcholine metabolism in low doses [86,87].

The inhibitory activity of *F. fomentarius* extracts in this study was superior to previously reported values for related mushroom species. For example, Orhan and Üstün [3] reported modest AChE inhibition (15 ± 1.82%) in 85% EtOH extracts of *F. fomentarius* from Turkey, and Dundar et al. [23] found little to no activity in other Turkish strains. Similarly, extracts of *Ganoderma lucidum* from Slovenia, including MeOH and EtOH extracts, exhibited lower activity compared to the MeOH extracts in this study [88]. Recent findings by Rašeta et al. [8] also highlight significant AChE inhibition in polar extracts of other fungal species from Serbia, further supporting the potential of *F. fomentarius* as a source of AChE inhibitors.

PCA revealed strong associations between the levels of PAs, TPC, TCC, and AChE inhibition (Figure 8). PC1 and PC2 explained 93.19% of the variance, with distinct clustering of the MeOH extracts in the first and third quadrants, alongside variables related to anti-AChE activity, DPPH, and FRAP assays. PUT and SPM clustered with anti-AChE activity, while SPD aligned with LP inhibition in the positive sections of both PCs. These patterns highlight the complementary roles of PAs, phenolic compounds, and carbohydrates in contributing to the neuroprotective potential of the extracts.

The MeOH extracts, particularly those from the FB strain, were rich in phenolic compounds such as quinic, *p*-hydroxybenzoic, cinnamic, *p*-coumaric, and caffeic acids, all of which have been identified as effective AChE inhibitors [89,90]. The higher TCC levels in the MeOH extracts also contribute to their observed activity, as carbohydrates and polysaccharides have been shown to play roles in neuroprotection and antioxidant activity [91,92].

The role of PAs in AChE inhibition remains an area of growing interest. Studies suggest that PAs such as SPD and SPM can either inhibit or stimulate AChE activity depending on the acetylcholine concentration [93]. Low doses of PAs have been shown to support memory enhancement and regulate NMDA receptors, while higher concentrations may result in pro-oxidative effects through hydrogen peroxide production [94]. Furthermore, SPD and SPM have demonstrated neuroprotective properties by modulating acetylcholine metabolism and receptor assembly [95,96].

### 3.4. Antiproliferative Activity

The antiproliferative potential of *F. fomentarius* extracts was assessed using the MTT assay on human cancer cell lines, including breast (MDA-MB-231, T47D, MCF7), cervical (HeLa, SiHa), and ovarian (A2780) cancer cells. Extracts prepared with different solvents from the FB strain showed varied effects on the cancer cells (Table 2).

The H_2_O extract had the mildest effect, with the most notable impact on the MCF-7 breast cancer cell line. Conversely, the CHCl_3_ extract showed more potent inhibition of cancer cell viability, especially affecting the T47D breast cancer line, while the MeOH and EtOH extracts, particularly from the FB strain, were identified as the most effective in inhibiting cancer cell growth.

The MeOH extracts of the FC and FS strains were further tested and found to significantly inhibit cancer cell viability, particularly in the A2780 (ovarian cancer) and MCF-7 (breast cancer) lines, although the FS extract generally exhibited stronger activity than the FC extract (Table 2). These results are in accordance with earlier studies, such as the study by Shnyreva et al. [97], who demonstrated that *F. fomentarius* MeOH–CHCl_3_ extracts exhibited antiproliferative activity against solid tumor cell lines.

In comparison with previous studies, the *F. fomentarius* extracts demonstrated higher activity against the HeLa cell line than reported by Dundar et al. [23], with their MeOH extract showing an IC_50_ value of 22.45 mg/mL. However, the results were lower compared to those reported by Kolundžić et al. [19], where cyclohexane, dichloromethane, MeOH, and H_2_O extracts exhibited an IC_50_ range of 8.31–51.87 μg/mL. These findings suggest that *F. fomentarius* extracts from Serbia might have a specific mechanism of action that selectively targets cancer cells while being non-toxic to normal cells, as demonstrated in a 2016 study [19].

The antiproliferative activity of these extracts correlated with their TPC and TCC levels, as presented in Figure S2. Flavonoids such as baicalein, chrysoeriol, and amentoflavone did not correlate with this activity, but coumarins like scopoletin and esculetin showed a weakly positive correlation. Cinnamic acid exhibited a negative correlation. However, other phenolic compounds, such as quinic acid, showed a moderate to high positive correlation with antiproliferative activity. Specifically, the MeOH FS extract, which had a high concentration of quinic acid, showed strong activity against the MDA-MB-231 breast cancer line, with a high correlation coefficient of 0.92.

Caffeic acid displayed the highest positive correlation (1.0) with the inhibition of MDA-MB-231 cell viability (Figure S1), highlighting its role as a potent antitumor agent, consistent with findings by Rosendahl et al. [98]. Conversely, flavonoids such as baicalein, chrysoeriol, and amentoflavone showed no significant correlation with antiproliferative activity, while coumarins (scopoletin and esculetin) demonstrated a weakly positive correlation. Interestingly, cinnamic acid exhibited a negative correlation with this activity.

The observed antiproliferative activity of the *F. fomentarius* extracts may be attributed to the synergistic effects of their bioactive components. Phenolic acids, particularly caffeic and quinic acids, have well-documented antitumor properties. For instance, quinic acid is recognized for its ability to enhance immune responses and induce apoptosis in cancer cells [77]. Caffeic acid is known to inhibit key signaling pathways involved in tumor progression [98].

Moreover, the selectivity of *F. fomentarius* extracts in targeting cancer cells while sparing normal cells is of particular interest for therapeutic applications. These findings align with recent studies highlighting the potential of fungal melanins from *F. fomentarius* to display protective and differentiative properties in neuroblastoma cells [76].

## 4. Conclusions

This study provides the first global documentation of polyamine levels in *F. fomentarius* and identifies six previously unreported phenolic compounds, including quinic acid. The extracts, particularly those obtained with methanol, exhibited strong neuroprotective and antiproliferative activities, highlighting the therapeutic relevance of this species. The significant acetylcholinesterase (AChE) inhibitory activity, especially in the polar extracts, suggests potential applications in the treatment of Alzheimer’s disease and other neurodegenerative conditions. Compounds such as quinic, *p*-hydroxybenzoic, and caffeic acids likely contributed to these effects, as indicated by their strong correlation with the observed bioactivity. Additionally, the extracts showed pronounced antiproliferative effects against several human cancer cell lines, particularly ovarian (A2780) and breast (MCF-7, MDA-MB-231) cancers. Their selectivity toward malignant cells, combined with low toxicity toward normal cells, positions *F. fomentarius* as a promising source of targeted anticancer agents, supporting its traditional medicinal use. These findings underscore the potential of *F. fomentarius* as a rich source of natural bioactive compounds for developing novel therapeutics for neurodegenerative diseases and cancer. Furthermore, its mineral content and antioxidant constituents support its use as a functional food supplement aimed at reducing oxidative stress, enhancing neuroprotection, and potentially lowering cancer risk. Continued evidence-based ethnopharmacological research is essential to validate these applications and facilitate its integration into modern medicine.

## Figures and Tables

**Table 1:** Concentrations of selected phenolic compounds determined using the LC-MS/MS method in the analyzed extracts of three different *Fomes fomentarius* strains.

Class	Compound	Amount of Selected Compound (μg/g d.w. ± SD)											
		*F. fomentarius* and Extract Type											
		FB				FC				FS			
		CHCl_3_	H_2_O	EtOH	MeOH	CHCl_3_	H_2_O	EtOH	MeOH	CHCl_3_	H_2_O	EtOH	MeOH
Flavones	Baicalein	<12.20	<12.20	<12.20	<12.20	<12.20	<12.20	<12.20	<12.20	<12.20	**147.50 ± 29.50 ^b^**	**44.63 ± 8.93 ^c^**	<12.20
	Chrysoeriol		<3.05	<3.05	<3.05	<3.05	<3.05	<3.05	<3.05	<3.05	<3.05	<3.05	<3.05
Biflavonoid	Amentoflavone	<3.05	<3.05	<3.05	<3.05	<3.05	<3.05	**10.97 ± 0.33 ^a^**	<3.05	<3.05	<3.05	<3.05	<3.05
Hydroxybenzoic acids	*p*-Hydroxybenzoic acid	<6.10	<6.10	**32.79 ± 1.97 ^c^**	**420.01 ± 25.20 ^a^**	<6.10	<6.10	<6.10	<6.10	<6.10	<6.10	<6.10	**56.03 ± 3.36 ^b^**
	Protocatechuic acid	<6.10	<6.10	**10.90 ± 0.87 ^c^**	**13.28 ± 1.06 ^b^**	<6.10	<6.10	<6.10	<6.10	<6.10	<6.10	<6.10	**25.48 ± 2.04 ^a^**
	Gentisic acid	<12.20	<12.20	<12.20	<12.20	<12.20	<12.20	<12.20	<12.20	<12.20	<12.20	<12.20	**22.46 ± 1.80 ^a^**
	Gallic acid	<12.20	<12.20	<12.20	<12.20	<12.20	<12.20	<12.20	<12.20	<12.20	<12.20	<12.20	<12.20
	Cinnamic acid	<97.50	<97.50	<97.50	<97.5	<97.50	<97.50	<97.50	<97.50	<97.50	<97.50	<97.50	<97.50
Hydroxycinnamic acids	*p*-Coumaric acid	<12.20	<12.20	<12.20	**83.12 ± 7.48 ^a^**	<12.20	<12.20	<12.20	<12.20	<12.20	<12.20	<12.20	**19.23 ± 1.73 ^b^**
	Caffeic acid	<6.10	<6.10	**22.18 ± 1.55 ^e^**	**83.36 ± 5.84 ^b^**	<6.10	<6.10	<6.10	<6.10	<6.10	**105.95 ± 7.42 ^a^**	**63.32 ± 4.43 ^c^**	**49.23 ± 3.45 ^d^**
Coumarins	Esculetin	<6.10	**51.36 ± 3.08 ^b^**	**73.66 ± 4.42 ^a^**	<6.10	<6.10	<6.10	<6.10	<6.10	<6.10	<6.10	<6.10	**84.03 ± 5.04 ^a^**
	Scopoletin	<6.10	<6.10	<6.10	**88.02 ± 7.04 ^e^**	<6.10	<6.10	<6.10	**23.63 ± 1.89 ^f^**	**209.10 ± 16.73 ^d^**	**511.70 ± 40.94 ^c^**	**853.07 ± 68.25 ^a^**	**580.80 ± 46.46 ^b^**
Cyclohexanecarboxylic acid	Quinic acid	<48.85	**58.99 ± 5.59 ^g^**	**414.20 ± 41.42 ^c^**	**996.80 ± 99.68 ^a^**	<48.85	**207.50 ± 20.75 ^f^**	**211.70 ± 21.17 ^f^**	**379.30 ± 37.93 ^d^**	<48.85	**247.80 ± 24.78 ^e^**	<48.85	**454.20 ± 45.42 ^b^**
Chlorogenic acid	5-*O*-caffeoylquinic acid	<6.10	<6.10	<6.10	<6.10	<6.10	<6.10	<6.10	<6.10	<6.10	<6.10	<6.10	**6.74 ± 0.34 ^a^**

Data represent the mean ± standard deviation (SD), and differences are considered statistically significant at *p* < 0.05. Different superscript letters (^a–g^) indicate significant differences (*p* < 0.05) according to Tukey’s HSD test (one-way ANOVA). **Bold number**: Amount of quantified phenolic compound in the examined extract. Values marked with the symbol “<” indicate concentrations below the LoQ (limit of quantification) but above the LoD (limit of detection), where the compound was detected but not quantifiable. The following abbreviations are used for the analyzed parameters: FB—strain sampled from Bosnia and Herzegovina; FC—strain sampled from Croatia; FS—strain sampled from Serbia; EtOH—hydroethanolic extract prepared with 70% EtOH; H_2_O—hot water extract; MeOH—hydromethanolic extract prepared with 80% MeOH CHCl_3_—chloroform extract.

**Table 2:** The antiproliferative activity of the selected *Fomes fomentarius* extracts.

Cell Line	Extract Concentration (μg/mL)	Growth Inhibition (% ± SEM)					
		FB				FC	FS
		H_2_O	EtOH	MeOH	CHCl_3_	MeOH	MeOH
MDA-MB-231	50	12.77 ± 3.30 ^d^	29.15 ± 1.58 ^c^	74.17 ± 0.34 ^a^	<10 *	12.83 ± 0.59 ^d^	46.55 ± 1.79 ^b^
	100	13.05 ± 2.57 ^d^	**89.06 ± 0.87 ^b^**	**94.98 ± 0.78 ^a^**	<10 *	51.39 ± 0.77 ^c^	**91.39 ± 0.83 ^b^**
MCF-7	50	29.17 ± 3.44 ^e^	65.84 ± 2.35 ^c^	85.23 ± 0.50 ^a^	38.14 ± 2.50 ^d^	60.99 ± 1.30 ^c^	75.88 ± 0.78 ^b^
	100	**70.97 ± 3.10 ^e^**	**91.34 ± 0.86 ^c^**	**95.96 ± 0.36 ^a^**	49.22 ± 1.52 ^f^	**80.84 ± 0.72 ^d^**	**93.42 ± 0.57 ^b^**
T47D	50	17.45 ± 2.08 ^e^	55.38 ± 0.72 ^b^	51.51 ± 1.50 ^cd^	49.19 ± 1.33 ^d^	53.06 ± 0.72 ^bc^	68.55 ± 0.94 ^a^
	100	20.72 ± 1.80 ^e^	**78.62 ± 0.83 ^b^**	**82.80 ± 1.58 ^a^**	**68.40 ± 1.19 ^c^**	63.59 ± 1.12 ^d^	**80.79 ± 0.90 ^ab^**
A2780	50	<10	**91.12 ± 0.43 ^c^**	**100.30 ± 0.36 ^a^**	25.07 ± 3.29 ^e^	69.20 ± 1.66 ^d^	**96.35 ± 0.72 ^b^**
	100	36.95 ± 3.79 ^c^	**100.50 ± 0.33 ^a^**	**99.94 ± 0.34 ^a^**	52.43 ± 0.99 ^b^	**100.20 ± 0.31 ^a^**	**99.21 ± 0.47 ^a^**
SiHa	50	16.44 ± 2.02 ^e^	66.97 ± 0.33 **^b^**	**93.13 ± 0.92 ^a^**	44.56 ± 1.62 ^d^	51.43 ± 2.86 ^c^	68.77 ± 0.31 **^b^**
	100	20.09 ± 1.58 ^d^	**95.42 ± 0.76 ^a^**	**95.75 ± 0.48 ^a^**	43.25 ± 1.82 ^c^	73.51 ± 1.26 **^b^**	**93.13 ± 1.02 ^a^**
HeLa	50	15.46 ± 2.47 ^e^	50.25 ± 2.04 ^c^	60.86 ± 3.10 **^b^**	36.87 ± 0.54 ^d^	62.75 ± 2.12 **^b^**	68.69 ± 1.35 **^a^**
	100	27.81 ± 1.07 ^e^	**92.80 ± 3.38 ^a^**	**96.31 ± 0.50 ^a^**	50.79 ± 1.50 ^d^	70.31 ± 2.96 ^c^	82.82 ± 2.48 **^b^**

Bolded values indicate the most promising tested extracts in terms of antiproliferative activity. * : <10: extracts exhibiting growth inhibition lower than 10% are considered ineffective and their data are not given numerically for clarity. Significant differences were determined by one-way ANOVA using Tukey’s HSD test. Data represent the mean ± SEM of two independent measurements, and differences were considered statistically significant at *p* < 0.05. Means within each row with different letters (a–f) are statistically significant. The following abbreviations are used for the examined parameters: FB—strain sampled from Bosnia and Herzegovina; FC—strain sampled from Croatia; FS—strain sampled from Serbia; d.w.—dry weight; CHCl_3_—chloroform extract; H_2_O—hot water extract; EtOH—hydroethanolic extract prepared with 70% EtOH; MeOH—hydromethanolic extract prepared with 80% MeOH; SEM—standard error of the mean; MDA-MB-231—human breast cancer cell line; MCF7—human breast cancer cell line; T47D—human breast cancer cell line; A2780—ovarian cancer cell line; SiHa—cervical cancer cell line; HeLa—cervical cancer cell line.

## Data Availability

All data are available within manuscript and the Supplementary Material.

## Supplementary Materials

The following supporting information can be downloaded at: https://www.mdpi.com/article/10.3390/microorganisms13061210/s1, Table S1: Optimized dynamic MRM parameters for all quantified compounds in the analyzed *F. fomentarius* extracts. Figure S1. LC-MS/MS chromatograms of the most abundant phenolic compounds and quinic acid in *Fomes fomentarius* extracts from three different Balkan localities. Strains are labeled as follows: FB—Bosnia and Herzegovina (blue); FC—Croatia (red); FS—Serbia (green). Identified phenolic compounds by extracts are: (**a**) CHCl_3_—chloroform extract: 1—scopoletin; 2—amentoflavone (Note: the CHCl_3_ extract of FB did not contain any phenolic compounds above the limit of quantification, LOQ); (**b**) H_2_O—hot water extract: 1—quinic acid; 2—caffeic acid; 3—esculetin; 4—scopoletin; 5—baicalein (Note: due to samples being analyzed at different times, a shift in retention times occurred, causing the peak of quinic acid (labeled as 1) to appear at two positions. However, the identification is reliable, as all samples were analyzed with calibration standards); (**c**) EtOH—70% hydroethanolic extract: 1—quinic acid; 2—protocatechuic acid; 3—*p*-hydroxybenzoic acid; 4—esculetin; 5—caffeic acid; 6—scopoletin; 7—baicalein (Note: due to samples being analyzed at different times, a retention time shift caused the peaks of quinic acid (1) and caffeic acid (5) to appear at two positions. The identification remains reliable due to calibration with standards; (**d**) MeOH—80% hydromethanolic extract: 1—quinic acid; 2—protocatechuic acid; 3—gentisic acid; 4—esculetin; 5—*p*-hydroxybenzoic acid; 6—caffeic acid; 7—*p*-coumaric acid; 8—scopoletin. Figure S2: Pearson’s correlation matrix for the examined parameters in three tested *F. fomentarius* strains, showing the relationships between total carbohydrate content (TCC), total phenolic content (TPC), quantified levels of 14 polyphenolics and quinic acid, and antiproliferative activity assessed using the MTT assay.

## Abbreviations

The following abbreviations are used in this manuscript:

FC	*Fomes fomentarius* strain sampled from Croatia
FB	*Fomes fomentarius* strain sampled from Bosnia and Herzegovina
FS	*Fomes fomentarius* strain sampled from Serbia
H_2_O	Hot water extract
EtOH	Hydroethanolic extract prepared with 70% ethanol
MeOH	Hydromethanolic extract prepared with 80% methanol
CHCl_3_	Chloroform extract
ROS	Reactive oxygen species
AChE	Acetylcholinesterase
AAS	Atomic absorption spectrophotometry
HPLC-FD	High-performance liquid chromatography coupled with fluorescence detector
LC-MS/MS	Liquid chromatography coupled with tandem mass-spectrometric detection
PA	Polyamine
PUT	Putrescine
SPD	Spermidine
SPM	Spermine
TPC	Total phenolic content
TCC	Total carbohydrate content
DPPH	2,2-Diphenyl-1-picrylhydrazyl radical, DPPH^•^
ABTS	2,2′-Azino-bis(3-ethylbenzothiazoline-6-sulfonic acid), ABTS^•+^
NO	Nitric oxide radical, NO^•^
FRAP	Ferric reducing antioxidant power
FC	Folin–Ciocalteu
AAE	Ascorbic acid equivalents
GAE	Gallic acid equivalents
GluE	Glucose equivalents
TE	Trolox equivalents
d.w.	Dry weight
dH_2_O	Distilled water
PG	Propyl gallate
SD	Standard deviation
SEM	Standard error of the mean
PCA	Principal component analysis
