# Supplementary material for: Bioactive Potential of Balkan Fomes fomentarius Strains: Novel Insights into Comparative Mycochemical Composition and Antioxidant, Anti-Acetylcholinesterase, and Antiproliferative Activities
Source: Microorganisms. 2025 May 26;13(6):1210. doi: 10.3390/microorganisms13061210 (PMC12195499; doi:10.3390/microorganisms13061210)
Supplement: Supplementary file 1 [file microorganisms-13-01210-s001.zip › microorganisms-3581842-supplementary.pdf]

microorganisms

MDPI

Table S1. Optimized dynamic MRM parameters for all quantified compounds in the analyzed *Fomes fomentarius* extracts.

Standard compounds	t_R [min]	Precursor *m*/*z*	Product *m*/*z*	$V_{fragmentor}$ (V)	$V_{collision}$ (V)
Gallic acid	0.61	169	125	90	10
p-Hydroxybenzoic acid	1.13	137	93	80	10
Protocatechuic acid	0.81	153	109	105	9
Gentisic acid	1.03	153	109	100	9
Caffeic acid	1.19	179	135	100	10
Cinnamic acid	3.91	147	103	100	5
p-Coumaric acid	1.73	163	119	90	9
Quinic acid	0.52	191	85	150	20
5-*O*-caffeoylquinic acid	0.80	353	191	100	10
Esculetin	1.15	177	133	105	15
Scopoletin	1.77	191	176	80	8
Amentoflavone	5.78	537	375	220	35
Baicalein	5.15	269	269	165	0
Chrysoeriol	4.82	299	284	125	20

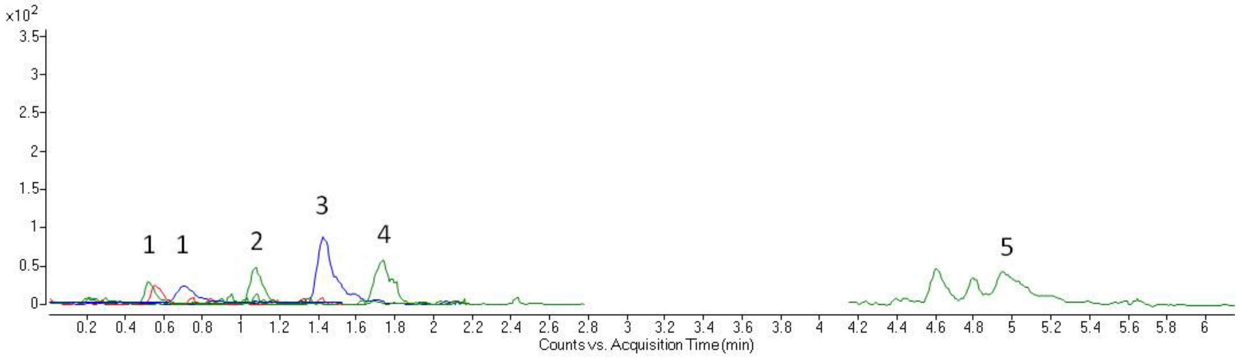

a)

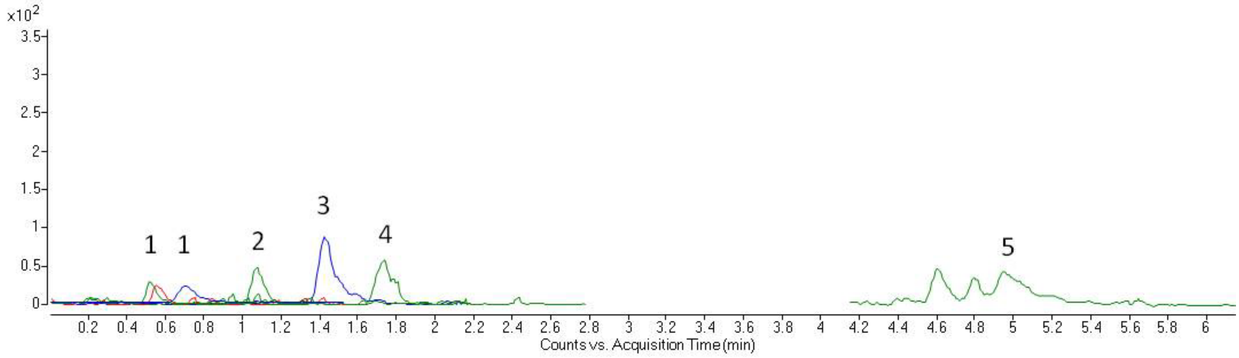

b)

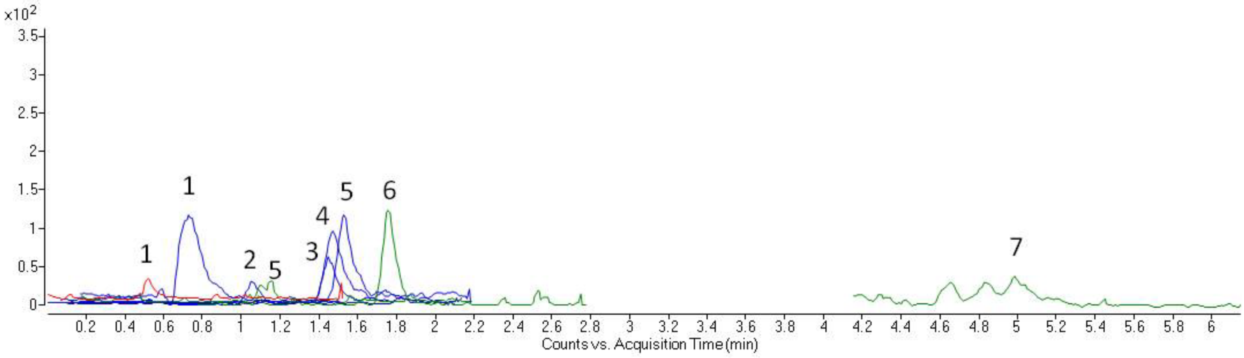

c)

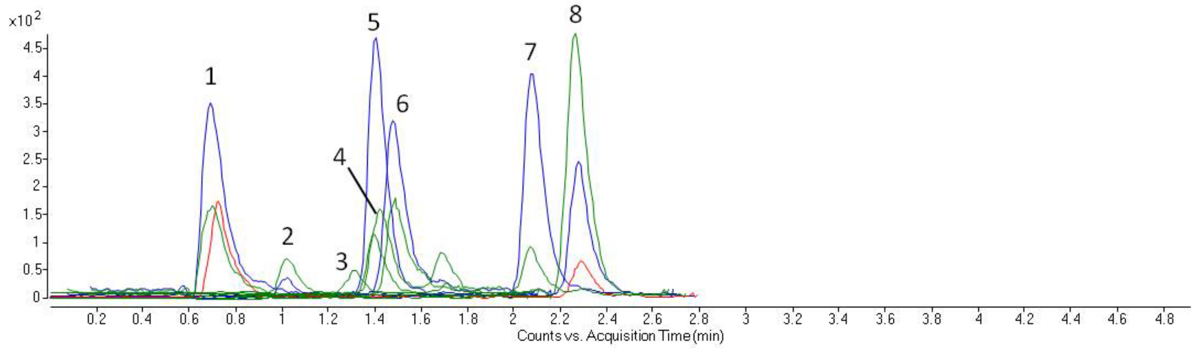

d)

Figure S1. LC-MS/MS chromatograms of the most abundant phenolic compounds and quinic acid in *Fomes fomentarius* extracts from three different Balkan localities. Strains are labeled as follows: FB

– Bosnia and Herzegovina (blue); FC – Croatia (red); FS – Serbia (green). Identified phenolic compounds by extracts are: (**a**) $CHCl_3$ – chloroform extract: 1 – scopoletin; 2 – amentoflavone (Note: the $CHCl_3$ extract of FB did not contain any phenolic compounds above the limit of quantification, LOQ); (**b**) H_2O – hot water extract: 1 – quinic acid; 2 – caffeic acid; 3- esculetin; 4 – scopoletin; 5 – baicalein (Note: due to samples being analyzed at different times, a shift in retention times occurred, causing the peak of quinic acid (labeled as 1) to appear at two positions. However, the identification is reliable, as all samples were analyzed with calibration standards); (**c**) EtOH – 70% hydroethanolic extract: 1 – quinic acid; 2 – protocatechuic acid; 3 – *p*-hydroxybenzoic acid; 4 – esculetin; 5 – caffeic acid; 6 – scopoletin; 7 – baicalein (Note: due to samples being analyzed at different times, a retention time shift caused the peaks of quinic acid (1) and caffeic acid (5) to appear at two positions. The identification remains reliable due to calibration with standards; (**d**) MeOH – 80% hydromethanolic extract: 1 – quinic acid; 2 – protocatechuic acid; 3 – gentisic acid; 4 – esculetin; 5 – *p*-hydroxybenzoic acid; 6 – caffeic acid; 7 – *p*-coumaric acid; 8 – scopoletin.

1		TCC	TPC	BA	CHR	AMEN	p-OH	PROTO	GENT	GA	CINN	p-CO	CAFF	ESC	SCO	QUI	5-O-CAF	MDA-MI	MDA-MI	MCF7 50	MCF7 10	T47D 50	T47D 10(	A2780 50	A2780 10	SiHa 50	SiHa 100	HeLa 50	HeLa 10(
2	TCC	1,00																											
3	TPC	0,78	1,00																										
4	BA	0,00	0,00	1,00																									
5	CHR	0,00	0,00	1,00	1,00																								
6	AMEN	0,00	0,00	1,00	1,00	1,00																							
7	p-OH	0,47	0,41	0,00	0,00	0,00	1,00																						
8	PROTO	0,54	0,43	0,00	0,00	0,00	0,24	1,00																					
9	GENT	0,62	0,34	0,00	0,00	0,00	-0,18	0,80	1,00																				
10	GA	0,45	0,23	0,00	0,00	0,00	-0,10	0,92	0,93	1,00																			
11	CINN	-0,58	-0,88	0,00	0,00	0,00	-0,14	-0,33	-0,31	-0,22	1,00																		
12	p-CO	0,47	0,38	0,00	0,00	0,00	1,00	0,22	-0,18	-0,10	-0,12	1,00																	
13	CAFF	0,60	0,53	0,00	0,00	0,00	0,90	0,63	0,19	0,32	-0,27	0,89	1,00																
14	ESC	-0,05	0,26	0,00	0,00	0,00	-0,33	0,61	0,47	0,62	-0,48	-0,37	0,01	1,00															
15	SCO	0,54	0,30	0,00	0,00	0,00	0,04	0,95	0,92	0,99	-0,25	0,03	0,44	0,57	1,00														
16	QUI	0,70	0,72	0,00	0,00	0,00	0,90	0,43	0,08	0,09	-0,40	0,88	0,91	-0,14	0,22	1,00													
17	5-O-CAFF	0,45	0,23	0,00	0,00	0,00	-0,10	0,92	0,93	1,00	-0,22	-0,10	0,32	0,62	0,99	0,09	1,00												
18	MDA-MB-231 50	0,59	0,58	0,00	0,00	0,00	0,90	0,63	0,18	0,30	-0,32	0,88	1,00	0,06	0,42	0,92	0,30	1,00											
19	MDA-MB-231 100	0,63	0,82	0,00	0,00	0,00	0,54	0,70	0,38	0,41	-0,56	0,50	0,76	0,36	0,49	0,83	0,41	0,80	1,00										
20	MCF7 50	0,81	0,80	0,00	0,00	0,00	0,61	0,59	0,41	0,33	-0,45	0,59	0,76	0,02	0,43	0,89	0,33	0,77	0,92	1,00									
21	MCF7 100	0,66	0,93	0,00	0,00	0,00	0,47	0,57	0,32	0,33	-0,90	0,44	0,64	0,47	0,40	0,71	0,33	0,69	0,84	0,71	1,00								
22	T47D 50	0,48	0,36	0,00	0,00	0,00	0,15	0,66	0,61	0,56	0,01	0,12	0,42	0,16	0,59	0,45	0,56	0,42	0,69	0,76	0,25	1,00							
23	T47D 100	0,38	0,33	0,00	0,00	0,00	0,42	0,54	0,30	0,31	0,09	0,39	0,59	0,00	0,38	0,64	0,31	0,59	0,75	0,80	0,27	0,92	1,00						
24	A2780 50	0,68	0,79	0,00	0,00	0,00	0,52	0,66	0,41	0,39	-0,47	0,49	0,73	0,23	0,47	0,83	0,39	0,76	0,98	0,97	0,75	0,79	0,83	1,00					
25	A2780 100	0,70	0,80	0,00	0,00	0,00	0,38	0,52	0,42	0,30	-0,47	0,35	0,55	0,12	0,36	0,74	0,30	0,58	0,90	0,95	0,66	0,81	0,80	0,96	1,00				
26	SiHa 50	0,56	0,57	0,00	0,00	0,00	0,74	0,55	0,19	0,22	-0,16	0,72	0,85	-0,05	0,33	0,90	0,22	0,86	0,88	0,91	0,55	0,74	0,90	0,91	0,83	1,00			
27	SiHa 100	0,60	0,72	0,00	0,00	0,00	0,48	0,62	0,37	0,35	-0,38	0,44	0,67	0,21	0,42	0,79	0,35	0,70	0,96	0,94	0,67	0,84	0,88	0,99	0,96	0,92	1,00		
28	HeLa 50	0,78	0,65	0,00	0,00	0,00	0,36	0,62	0,60	0,48	-0,28	0,34	0,57	0,02	0,55	0,68	0,48	0,57	0,79	0,93	0,49	0,91	0,84	0,89	0,93	0,81	0,89	1,00	
29	HeLa 100	0,53	0,68	0,00	0,00	0,00	0,55	0,54	0,24	0,23	-0,31	0,51	0,70	0,12	0,31	0,82	0,23	0,73	0,94	0,93	0,63	0,79	0,90	0,97	0,93	0,95	0,99	0,85	1,00

Figure S2. Pearson's correlation matrix for the examined parameters in three tested *Fomes fomentarius* strains, showing the relationships between total carbohydrate content (TCC), total phenolic content (TPC), quantified levels of 14 polyphenolics and quinic acid, and antiproliferative activity assessed using the MTT assay. Green squares represent a highly significant correlation of inspected parameters, while red squares present low interactions, assessed according to the corresponding Pearson's coefficient. Here are the abbreviations for the parameters analyzed: TCC – total carbohydrate content; TPC – total phenolic content; BA – baicalein; CHR – chrysoeriol; AMEN – amentoflavone; p-OH – *p*-hydroxybenzoic acid; PROTO – protocatechuic acid; GENT – gentisic acid; GA – gallic acid; CINN – cinnamic acid; *p*-CO – *p*-coumaric acid; CAFF – caffeic acid; ESC – esculetin; SCO – scopoletin; QUI – quinic acid; 5-O-CAFF – 5-*O*-caffeoylquinic acid; 50 – extract was applied in concentration of 50 μg/mL; 100 – extract was applied in concentration of 100 μg/mL; MDA-MB-231 – human breast cancer cell line; MCF7 – human breast cancer cell line; T47D – human breast cancer cell line; A2780 – ovarian cancer cell line; SiHa – cervical cancer cell line; HeLa – cervical cancer cell line.
